# A New Presenilin 1 (Psen1) Mutation (p.Cys263Trp) as a Cause of Both Early and Late-Onset Alzheimer’s Disease in a Large Italian Family

**DOI:** 10.3390/ijms22126215

**Published:** 2021-06-09

**Authors:** Rosanna Tortelli, Davide Seripa, Chiara Zecca, Maria Teresa Dell’Abate, Paola Bisceglia, Maria Rosaria Barulli, Roberto De Blasi, Giancarlo Logroscino

**Affiliations:** 1 Center for Neurodegenerative Diseases and the Aging Brain, University of Bari “Aldo Moro”—A.O. Pia Fondazione Cardinale G. Panico, 73039 Tricase, Italy; chiarazecca.cz@gmail.com (C.Z.); dellabatemariateresa@gmail.com (M.T.D.); orietta.barulli@gmail.com (M.R.B.); robertodeblasi@hotmail.com (R.D.B.); 2 Complex Unit of Geriatrics, Department of Medical Sciences, IRCCS Casa Sollievo della Sofferenza, 71013 San Giovanni Rotondo, Italy; dseripa@gmail.com (D.S.); pabis88@hotmail.it (P.B.); 3 Laboratory for Advanced Hematological Diagnostics, Department of Hematology and Stem Cell Transplant, “Vito Fazzi” Hospital, 73100 Lecce, Italy; 4 Department of Radiology, “Pia Fondazione Cardinale G. Panico”, 73039 Tricase, Italy; 5 Department of Basic Medicine, Neuroscience, and Sense Organs, University of Bari ‘Aldo Moro’, 70124 Bari, Italy

**Keywords:** Alzheimer’s disease, PSEN1 mutations, phenotype heterogeneity

## Abstract

Mutations in the PSEN1 gene are the most common cause of autosomal dominant Alzheimer’s disease, and are characterized by a high phenotype variability. This study describes a five-generation family, with a prevalent late-onset of the disease and a high frequency of depression, in which a new missense mutation (c.789T > G, p.Cys263Trp) in exon 8 of the PSEN1 gene was found. Only the proband presented an early onset at the age of 45 with attention deficit, followed by spatial disorientation, psychiatric symptoms and parkinsonian signs. The other two cases had a late onset of the disease and a typical presentation with memory loss. Both were characterized by a high level of anxiety and depression. The disease course was different with signs of Lewy body dementia for the proband’s mother, and pyramidal involvement and a shorter disease duration for the proband’s maternal aunt. The other eight cases with late-onset dementia and three cases with a long history of depression have been reported in the family pedigree, underlying the high phenotype variability of PSEN1 mutations.

## 1. Introduction

It has been estimated that in 2016, the global number of individuals living with dementia was 43.8 million, worldwide, increased from 20.2 million in 1990 [1], and expected to be around 100 millions in 2050 [2]. Alzheimer’s disease (AD) is the leading cause of neurodegenerative dementia. Based on the age of onset (AoO), AD encompasses an early-onset form, with an onset before 65 years of age (EOAD), and a late-onset phenotype (LOAD). The majority of AD cases are sporadic, with a not well-defined etiology. However, a small proportion of AD cases, especially among EOAD, are reported to be familial with an autosomal dominant pattern of inheritance (ADAD) [3]. These cases are due to three main causative genes: amyloid precursor protein (APP) [4], presenilin 1 (PSEN1) [5], and presenilin 2 (PSEN2) [6], all linked to the amyloid-cascade hypothesis. Mutations in the PSEN1 gene are the most common cause of ADAD [7], with more than 300 different mutations currently described worldwide (www.molgen.ua.ac.be/ADMutations, accessed on 15 January 2021; http://www.alzforum.org/mutations, accessed on 15 January 2021).

PSEN1 pathogenic variations have been linked to heterogeneous phenotypes, ranging from classical AD with a prevalent memory impairment and a very early AoO (3rd–4th decades), to atypical presentations with prominent and early behavioral impairment, psychiatric manifestations, spastic paraparesis, extrapyramidal signs, ataxia, seizures and myoclonus [8]. It has been hypothesized that the phenotypic variability, also within the same family, can be due to the distribution of the lesions in different cortical areas [9].

In the present study, we described for the first time a new PSEN1 mutation in a five-generation family, with a high phenotype heterogeneity.

## 2. Results

**Figure 1:**
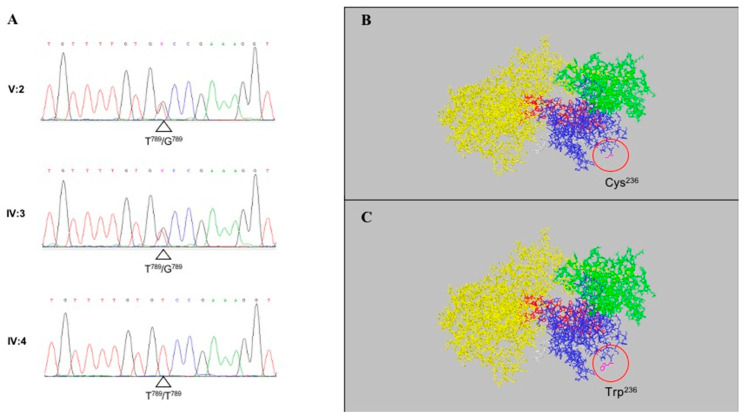
Panel (**A**) sequencing data showing the T-to-G missense mutation in exon 8 (c.789T > G). Panels (**B**,**C**) 3D model of the native (panel (**B**)) and mutated (panel (**C**)) γS complexes. Nicastrin protein is presented in yellow, PSEN1 in blue, the APH-1A subunit in green, PSEN2 in red, the poly-Ala chain in grey, and the aminoacid 263 in violet.

The family pedigree is reported in Figure 1. The proband (V:2) was a 48-year old right-handed male, with 13 years of education, who was referred to our center with a 3-year history of cognitive decline. He worked as a painter. In his medical history there was a meningococcal meningitis at the age of 11, which did not result in any neurological deficit. No other diseases were mentioned. At the age of 45 he started to experience a reduction in attention and concentration capacities, an impairment in planning abilities and ideomotor apraxia with working difficulties until he was forced to stop his job at the age of 47. During the same period he started to manifest behavioral changes with apathy, disinhibition, poor personal hygiene and irritability. This was interpreted as being due to depression and anxiety because of personal and family problems and he was treated with antidepressants with no efficacy. Memory loss appeared almost one year later, followed by episodes of spatial disorientation. At admission, neurological examination was normal. Neuropsychological assessment revealed a moderate dementia with predominant involvement of memory (verbal and visual) and executive functions; loss of insight and ideomotor apraxia were also present. His mini mental state examination (MMSE) was 14/30, Clinical Dementia Rating Scale—Global Score (CDR-GS) was 2.0 (moderate dementia). The brain MRI showed only a slight diffuse cortical atrophy, without any specific pattern. Cerebrospinal fluid analysis revealed a specific AD pattern with decreased Aβ_1–42_ level (298 pg/mL; reference range: 610–974 pg/mL), and increased t-Tau (605 pg/mL; reference range: 47–225 pg/mL) and p181-Tau (75.2 pg/mL; reference range: 35.84–66.26 pg/mL). A diagnosis of “Probable AD dementia with evidence of AD pathophysiological process” was made [10]. The cognitive decline progressed rapidly, with complete loss of autonomy in activities of daily living in five years. At this time, he developed moderate rigidity of the trunk and of the four limbs, bradykinesia and camptocormia. He presented also visual hallucinations and delusions. His irritability and aggressive behavior became more frequent and severe and caused institutionalization. After six years from symptom onset, he developed fast progressive dysphagia and underwent percutaneous endoscopic gastrostomy in 1 year.

The proband’s mother (IV:3) was a 70 year old right-handed woman with 5 years of education. At the age of 63 she developed depression and anxiety, not improved by antidepressant intake. At the age of 68 she started to manifest memory impairment and word-finding difficulties. Almost one year later she developed temporal and spatial disorientation, and she started presenting marked impairment in planning daily activities and in logical reasoning. When she came to our attention, the neurological examination did not reveal any motor, sensory or cranial-nerve alteration. The neuropsychological examination revealed severe memory loss, impairment of visuospatial abilities, ideomotor and dressing apraxia. CDR-GS score was 2.0 (moderate dementia). MMSE score was 15/30. She was not able to perform a brain MRI scan because of claustrophobia. The brain CT scan showed moderate diffuse cortical atrophy. Electroencephalography revealed the presence of theta waves on frontotemporal regions bilaterally. She refused to undergo lumbar puncture. A diagnosis of “Probable AD dementia, based on clinical criteria” was made [10]. After one year of follow-up, her anxiety sharply increased with panic attacks and claustrophobia, hardly controlled by pharmacological therapy. She became emotionally dependent on her husband. Furthermore, she started to manifest visual hallucinations, arousal fluctuations and right hand dystonia, followed by the appearance of bradykinesia, slight symmetrical limb rigidity and camptocormia. Her walking ability sharply decreased over the following three years and she became wheelchair bound. She developed severe dementia with complete loss of autonomy in nine years from symptom onset.

The proband’s maternal aunt (IV:5) came to our attention at the age of 74, with a long history of depression and a 2-year history of memory loss. She was right-handed and with 8 years of education. Her personal history was remarkable for atrial fibrillation, a mitral mechanical substitution, essential hypertension, chronic kidney failure, and thyropathy. A neuropsychological evaluation revealed prominent memory loss, and mild executive dysfunction. Her CDR-GS score was 1.0 (mild dementia), MMSE score was 18/30. A brain MRI was not performed because of the mechanical cardiac valve. The brain CT scan showed mild, diffuse fronto-parietal atrophy. She refused to undergo lumbar puncture. Based on clinical phenotype and on the results of genetic testing, a diagnosis of “Probable AD dementia in a carrier of a causative AD genetic mutation” was made, and the same diagnosis was adopted at this time for the other two examined family members. She experienced a rapid cognitive decline with almost complete loss of independence in four years. At the last neurological examination she had gait difficulties, slight bilateral leg spasticity along with increased deep tendon reflexes. 

Genetic analysis of the PSEN1 gene revealed a new T-to-G missense mutation in exon 8 (c.789T > G) (Figure 1A), resulting in the substitution at codon 263 of a cysteine (TGT) with a tryptophane (TGG) (p.Cys263Trp). Three dimensional models of the native (Cys263) and mutated (Trp263) complexes are reported in Figure 1B,C, respectively. The APOE haplotype of the proband was e3/e3. This information was not available for the other two family members. 

We also sequenced the PSEN1 gene in one healthy control (proband’s father; IV:4) and the missense mutation was not present. 

**Figure 2:**
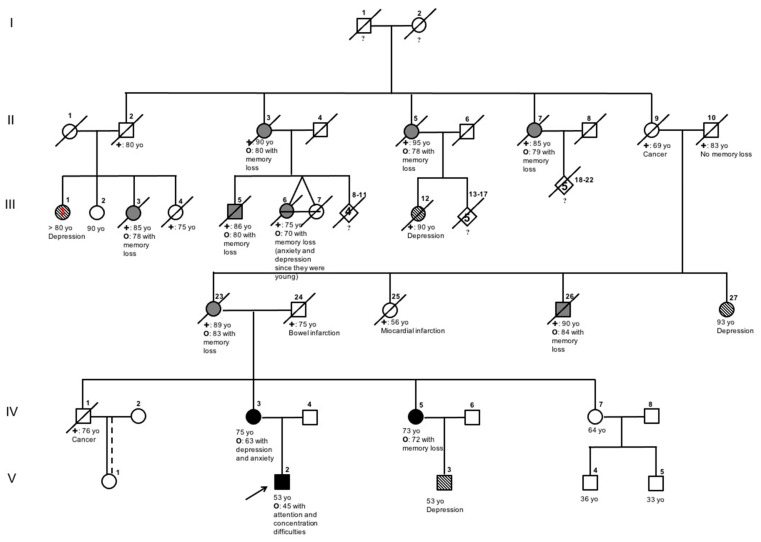
Family pedigree. Symbols: roman numbers to the left of the pedigrees denote generations. The arrow indicates the proband. Circles indicate females and squares indicate males. Fully black symbols indicate the examined affected individuals. Fully gray symbols denote the individuals reported as affected by the informants. Striped symbols denote personal history of depression.

The family pedigree (Figure 2) showed an autosomal dominant pattern of inheritance, spreading on V generations. All the affected cases presented a late AoO of the disease (all over 70 years of age) with memory loss (II:3, II:5, II:7, III:3, III:5, III:6, III:23, III:26). A family history of late- and early-onset depression was also reported (III:1, III:12, III:27, V:3).

## 3. Discussion

In the present study, we reported a new Cys^263^ →Trp substitution in the PSEN1 gene in three cases of the same family with both early onset and late-onset AD, and a high phenotype heterogeneity. 

The other two mutations on the same codon have been described already [11,12]. Wasco and coworkers reported a missense mutation which resulted in the substitution of a cysteine for an arginine (C263R) [12], whereas Janssen and colleagues described the C263F (cysteine/phenylalanine) substitution among seven novel PSEN1 point mutations [11]. They were not able to demonstrate the segregation of the variant with the disease, but proposed it as pathogenic, given the previous description of the other pathogenic mutation on the same codon [11]. In our work, segregation is confirmed by the presence of the mutation in three affected family members over two generations and the absence of the same mutations in the unaffected proband’s father. Furthermore, since both proband’s mother and maternal aunt presented the same variation, it is very unlikely that this is a de novo mutation, but is likely to be transmitted through generations inside the family pedigree. 

The pathogenicity of the present mutation is also supported by the type of amino acid substitution (a polar amino acid with a small tiol side chain, with a far less polar amino acid with a big indole side chain). The different steric size of the two could be responsible for the altered tertiary and quaternary conformations of the protein. Interestingly, some authors reported the position of this amino acid at the end of the sixth transmembrane hydrophobic domain [13], whereas others at the beginning of the cytoplasmatic loop [14]. This can influence the effect of the amino acid substitution in this position. Notably, given the great variability of the cytoplasmatic loops, their reconstruction is missing in the available structure model (https://www.ncbi.nlm.nih.gov/structure/, 15 January 2021), limiting the tridimensional reconstruction of the γS complex to the trans-membrane domains. 

The majority of PSEN1 mutations show complete penetrance by the age of 60, with a prodromal phase of the disease beginning as early as 30 years of age [15]. However, a wide age range has been described for some PSEN1 pathogenic variants [16,17,18]. It is recognized that the type of mutation and the parent’s AoO account for a substantial proportion of the AoO variability, but a large residual variation is still observed between and within families with the same pathogenic mutation [17]. The mutations on the same codon manifested with an early AoO, between 47 and 60 years of age [11,12]. In a recent study Ryan and colleagues identified mutations located before codon 200 as associated to EOAD, and those after codon 200 more likely to cause a late onset of the disease [8]. However, they commented on some exceptions to this rule, since a young onset of the disease has been described for several mutations located after codon 200 (L226F, M233I, M233T, L232P, L235P, and L248P)., and proposed to slightly shift the generalized threshold for EOAD mutations towards the C-terminal region of PSEN1 [8]. Apart from the proband, our pedigree presents an atypical, but fairly homogeneous phenotype in terms of AoO. It is extremely rare to have such a late-onset for monogenic AD and only two other cases with an onset around the age of 70 have been described [19,20]. Regarding the early AoO of the proband, it is likely that factors other than the type and site of mutation might have influenced the phenotype. Sepulveda-Falla described a 30-year range of AoO (39–70 years) for the E280A mutation in a large Colombian pedigree of more than 5000 subjects and more than 1000 carriers [18]. They identified different patterns of hyperphosphorylated tau (pTau) pathology, as well as alterations in kinase activity, as the main determinants of AoO variability, with a high pathology burden, diffuse aggregation pattern, and activation of stress kinases in individuals with an early age of onset [18]. It is reasonable to think that similar determinants, as well as other unknown genetic modifiers could have been responsible for the phenotype switch observed in the proband. 

The phenotype heterogeneity of our pedigree implicated factors other than AoO. The proband presented a more frontal onset of the disease, characterized by behavioral symptoms (apathy, disinhibition, personality changes, and irritability), associated with a dysexecutive syndrome (impairment of planning abilities). During the course of the disease he also showed frank psychotic symptoms, namely visual hallucinations and delusions and a worsening of his irritability, which became resistant to pharmacological therapy. Parkinsonian signs were also present in the advanced stage of the disease. The other two examined cases, with a late onset of the disease, showed a phenotype dominated by negative psychiatric symptoms, namely depression and anxiety, which had preceded by many years the onset of the disease and continued to be prominent during the subsequent course. The disease had a typical onset with memory impairment, while atypical symptoms appeared at a later stage. The proband’s mother developed structured visual hallucinations, arousal fluctuations and parkinsonism, resembling a phenotype of Lewy bodies dementia. The proband maternal aunt presented pyramidal motor impairment. The three examined cases also differed in terms of disease duration from symptom onset to complete loss of independence, which was faster for the proband’s maternal aunt (4 years), compared to the proband (5 years) and the proband’s mother (9 years). PSEN1 mutations are more frequently associated with phenotype heterogeneity and atypical presentation, compared to APP and PSEN2 mutations [8]. A behavioral/dysexecutive onset has been described in around 10% of PSEN1 mutations [8,21], and an atypical cognitive presentation, accompanied by pyramidal signs, has been associated to mutations in exon 8 [8], as in our case series. Mutations in exon 8 have also been associated with diffuse white-matter hyperintensities at the brain MRI scan, along with signs of amyloid angiopathy [22,23]. It has been hypothesized that this could be related to the fact that exon 8 encodes residues of the hydrophilic loop between transmembrane domains 6 and 7, that is the site of cleavage of APP, but also of other substrates, which could contribute to pathological alterations other than amyloid deposition (white matter hyperintensities) and, eventually, to the atypical features of the disease [8,24]. However, in our case series, the proband was the only subject able to undergo a brain MRI scan, which only showed a slight diffuse cortical atrophy. 

Another main phenotypic trait of the described pedigree was the presence of a depressive disorder in both affected and unaffected individuals. The tight link between depression and AD is largely recognized and a prodromal role for depression has been hypothesized [25]. A depressive disorder has already been described as preceding by 10–15 years the cognitive decline in a family pedigree with another PSEN1 mutation (Leu173Phe) [26]. The presence of depression in other family members not affected by dementia (as referred by the informants) might be supportive of a prodromal role of depression in the phenotypic manifestation of this mutation. We can also speculate an incomplete penetrance, rarely reported for some PSEN1 mutations [27]. Interestingly, higher levels of depression have already been described in female preclinical PSEN1 mutation carriers, compared to non-carriers, unaware of their genetic status [28].

Several limitations must be mentioned for our study. Firstly, no functional studies have been performed to examine the mechanistic effect of the mutation on AD pathogenesis. Secondly, we were not able to perform genetic analysis on one unaffected family member (internal control) in order to conduct a proper segregation analysis. However, the finding of the same mutation in three affected family members over two generations, as long as the position of the mutation and the in silico prediction of its effect on the protein structure, corroborate its pathogenic role. Furthermore, the lack of pathological confirmation of the disease should also be mentioned, as well as the availability of CSF biomarkers for only one of the examined patients, and the lack of specific positron emission tomography biomarkers for all of them. 

In conclusion, this study identified a novel mutation in exon 8 of the PSEN1 gene, in a five-generation family pedigree with a high phenotype variability. Our study expands the PSEN1 phenotype, especially in terms of AoO, strengthening the link between monogenic forms and LOAD, and the role of depression as a possible prodromal phase of the disease. Further studies are needed to elucidate possible phenotype modifiers in order to increase precision in patients stratification for future clinical trials. 

## 4. Materials and Methods

### 4.1. Clinical and Paraclinical Assessment

Patients were assessed and followed-up at the Center for Neurodegenerative Diseases and the Aging Brain, University of Bari “Aldo Moro”—A.O. Pia Fondazione Cardinale G. Panico, Tricase (LE), Italy. As part of the diagnostic process, examined patients underwent a detailed clinical assessment, including personal medical history, standard neurological examination, and cognitive evaluation, including the Clinical Dementia Rating Scale (CDR), the mini mental state examination (MMSE) [29], and a complete neuropsychological assessment, using a battery of cognitive tests, described already [30]. As per diagnostic protocol, patients underwent routine blood biochemical analyses, a high-field (3-Tesla) brain magnetic resonance imaging (MRI) scan, and a cerebrospinal fluid collection, through a lumbar puncture, for biomarker analysis. Measures of AD related CSF biomarkers, Aβ_1–42_, total-tau (t-Tau), and phosphorylated 181-Tau (p181-Tau) was made using commercially available ELISA immunoassays (Innotest β-amyloid1–42, Innotest hTau-Ag, Innotest Phospho Tau (181P), Fujirebio Europe N.V., Gent, Belgium). All the assays were performed according to manufacturer’s protocols. After a written informed consent had been signed, a blood sample was also collected for genetic analyses and shipped to the Genetic Laboratory, Complex Unit of Geriatrics, Department of Medical Sciences, IRCCS Casa Sollievo della Sofferenza, San Giovanni Rotondo (FG), Italy. Clinical diagnosis was made according to the National Institute of Aging and Alzheimer’s Association (NIA-AA) criteria for Alzheimer’s disease [10]. 

A detailed family history was collected for the examined patients. Family pedigree was reconstructed and drawn, based on the information collected from the examined patients, one caregiver (proband’s father; IV:4), who accepted to undergo genetic analysis as external control, and one unaffected family member (IV:7), who refused herself to undergo genetic analysis as internal control. No other family members, but the three examined cases, were available for genetic analysis. 

This study was waived from ethical approval, since all the procedures were done as part of clinical assessment and participants signed a written informed consent about the anonymized use of their data for research.

### 4.2. Genetic Analysis

Genomic DNA was extracted from fresh or frozen blood samples using the salting-out method [31]. PCR was performed with 50 ng of genomic DNA, 10 pmol of each primer (5′-TTACAAGTTTAGCCCATACATTTT-3′ and 5′-TCAGTTCCGATAAATTCTAC-3′ for exon 8 PSEN1) and 5 U/µL of Platinum Taq DNA polymerase (Invitrogen, Carlsbad, CA, USA) in the following conditions: 2 min at 94 °C, followed by 35 cycles of steps at 0.45 s. at 94 °C, 0.45 s. at 58 °C, and 0.45 s, at 72 °C. PCR products were purified with ExoSAP-IT (Affymetrix, Santa Clara, CA, USA) and sequenced in both directions using BigDye terminator v1.1 chemistry on an ABI PRISM 3130XL Genetic Analyzer (Applied Biosystems, Austin, TX, USA). The 3D model of the γS complex was made with the Swiss-PdbViewer Ver. 4.1.0 software by using the 5FN3 structure model [32].

## References

[1] GBD 2016 Demmentia Collaborators (2019). Global, regional, and national burden of Alzheimer’s disease and other dementias, 1990–2016: A systematic analysis for the Global Burden of Disease Study 2016. Lancet Neurol..

[2] Brookmeyer R., Johnson E., Ziegler-Graham K., Arrighi H.M. (2007). Forecasting the global burden of Alzheimer’s disease. Alzheimers Dement..

[3] Cacace R., Sleegers K., van Broeckhoven C. (2016). Molecular genetics of early-onset Alzheimer’s disease revisited. Alzheimers Dement..

[4] Goate A., Chartier-Harlin M.-C., Mullan M., Brown J., Crawford C., Fidani L., Giuffra L., Haynes A., Irving N., James L. (1991). Segregation of a missense mutation in the amyloid precursor protein gene with familial Alzheimer’s disease. Nature.

[5] Sherrington R., Rogaev E.I., Liang Y., Rogaeva E.A., Levesque G., Ikeda M., Chi H., Lin C., Li G., Holman K. (1995). Cloning of a gene bearing missense mutations in early-onset familial Alzheimer’s disease. Nature.

[6] Rogaev E.I., Sherrington R., Liang Y., Rogaeva E.A., Levesque G., Ikeda M., Chi H., Lin C., Li G., Holman K. (1995). Familial Alzheimer’s disease in kindreds with missense mutations in a gene on chromosome 1 related to the Alzheimer’s disease type 3 gene. Nature.

[7] Raux G., Guyant-Maréchal L., Martin C., Bou J., Penet C., Brice A., Hannequin D., Frebourg T., Campion D. (2005). Molecular diagnosis of autosomal dominant early onset Alzheimer’s disease: An update. J. Med. Genet..

[8] Ryan N.S., Nicholas J.M., Weston P.S.J., Liang Y., Lashley T., Guerreiro R., Adamson G., Kenny J., Beck J., Chavez-Gutierrez L. (2016). Clinical phenotype and genetic associations in autosomal dominant familial Alzheimer’s disease: A case series. Lancet Neurol..

[9] Bruni A.C., Bernardi L., Colao R., Rubino E., Smirne N., Frangipane F., Terni B., Curcio S.A.M., Mirabelli M., Clodomiro A. (2010). Worldwide distribution of PSEN1 Met146Leu mutation: A large variability for a founder mutation. Neurology.

[10] McKhann G.M., Knopman D.S., Cherthow H., Hyman B.T., Jack C.R., Kawas C.K., Klunk W.E., Koroshetz W.J., Manly J.J., Mayeux R. (2011). The diagnosis of dementia due to Alzheimer’s disease: Recommendations from the National Institute on Aging-Alzheimer’s Association workgroups on diagnostic guidelines for Alzheimer’s disease. Alzheimers Dement..

[11] Janssen J.C., Beck J.A., Campbell C.A., Dickinson A., Fox N.C., Harvey R.J., Houlden H., Rossor M.N., Collinge J. (2003). Early onset familial Alzheimer’s disease: Mutation frequency in 31 families. Neurology.

[12] Wasco W., Pettingell W.P., Jondro P.D., Schmidt S.D., Gurubhagavatula S., Rodes L., DiBlasi T., Romano D.M., Guenette S.Y., Kovacs D.M. (1995). Familial Alzheimer’s chromosome 14 mutations. Nat. Med..

[13] Hardy J. (2007). Putting presenilins centre stage. Introduction to the Talking Point on the role of presenilin mutations in Alzheimer disease. EMBO Rep..

[14] Fraser P.E., Yang D.-S., Yu G., Levesque L., Nishimura M., Arawaka S., Serpell L.C., Rogaeva E., St George-Hyslop P. (2000). Presenilin structure, function and role in Alzheimer disease. Biochim. Biophys. Acta.

[15] Larner A.J., Doran M. (2006). Clinical phenotypic heterogeneity of Alzheimer’s disease associated with mutations of the presenilin-1 gene. J. Neurol..

[16] Axelman K., Basun H., Lannfelt L. (1998). Wide range of disease onset in a family with Alzheimer disease and a His163Tyr mutation in the presenilin-1 gene. Arch. Neurol..

[17] Ryman D.C., Acosta-Baena N., Aisen P.S., Bird T., Danek A., Fox N.C., Goate A., Frommelt P., Ghetti B., Langbaum J.B.S. (2014). Symptom onset in autosomal dominant Alzheimer disease: A systematic review and meta-analysis. Neurology.

[18] Sepulveda-Falla D., Chavez-Gutierrez L., Portelius E., Velez J.I., Dujardin S., Barrera-Ocampo A., Dinkel F., Hagel C., Puig B., Mastronardi C. (2021). A multifactorial model of pathology for age of onset heterogeneity in familial Alzheimer’s disease. Acta Neuropathol..

[19] Scarioni M., Arighi A., Fenoglio C., Sorrentino F., Serpente M., Rotondo E., Mercurio M., Marrota G., Dijkstra A.A., Pijnenburg Y.A.L. (2020). Late-onset presentation and phenotypic heterogeneity of the rare R377W PSEN1 mutation. Eur. J. Neurol..

[20] Zhang S., Li X., Xhang L., Meng X., Ma L., Zhang G., Wu H., Liang L., Cao M., Mei F. (2020). Identification of a Rare PSEN1 Mutation (Thr119Ile) in Late-Onset Alzheimer’s Disease With Early Presentation of Behavioral Disturbance. Front. Psychiatry.

[21] Wallon D., Rousseau S., Rovelet-Lecrux A., Quillard-Muraine M., Martinaud O., Pariente J., Puel M., Rollin-Sillaire A., Pasquier F., Le Ber I. (2012). The French series of autosomal dominant early onset Alzheimer’s disease cases: Mutation spectrum and cerebrospinal fluid biomarkers. J. Alzheimers Dis..

[22] Mann D.M., Pickering-Brown S.M., Takeuchi A., Iwatsubo T., Members of the Alzheimer’s Disease Pathology Study Group (2001). Amyloid angiopathy and variability in amyloid beta deposition is determined by mutation position in presenilin-1-linked Alzheimer’s disease. Am. J. Pathol..

[23] Ryan N.S., Biessels G.-J., Kim L., Nicholas J.M., Barber P.A., Walsh P., Gami P., Morris H.R., Beck J., Mead S. (2015). Genetic determinants of white matter hyperintensities and amyloid angiopathy in familial Alzheimer’s disease. Neurobiol. Aging.

[24] Chávez-Gutiérrez L., Bammens L., Benilova I., Vandersteen A., Benurwar M., Borgers M., Lismont S., Zhou L., Karran E., Gijsen H. (2012). The mechanism of γ-Secretase dysfunction in familial Alzheimer disease. EMBO J..

[25] Agüera-Ortiz L., Garsia-Ramos R., Grandas Perez F.J., Lopez-Alvarez J., Montes Rodriguez J.M., Olazaran Rodriguez F.J., Pueyo J.O., Valero C.P., Porta-Etessam J. (2021). Depression in Alzheimer’s Disease: A Delphi Consensus on Etiology, Risk Factors, and Clinical Management. Front. Psychiatry.

[26] Kasuga K., Ohno T., Ishihara T., Miyashita A., Kuwano R., Onodera O., Nishizawa M., Ikeuchi T. (2009). Depression and psychiatric symptoms preceding onset of dementia in a family with early-onset Alzheimer disease with a novel PSEN1 mutation. J. Neurol..

[27] Rossor M.N., Fox N.C., Beck J., Campbell T.C., Collinge J. (1996). Incomplete penetrance of familial Alzheimer’s disease in a pedigree with a novel presenilin-1 gene mutation. Lancet.

[28] Ringman J.M., Diaz-Olavarrieta C., Rodriguez Y., Chavez M., Paz F., Murrell J., Angel Macias M., Hill M., Kawas C. (2004). Female preclinical presenilin-1 mutation carriers unaware of their genetic status have higher levels of depression than their non-mutation carrying kin. J. Neurol. Neurosurg. Psychiatry.

[29] Folstein F.M., Folstein S.E., McHugh P.R. (1975). “Mini-mental state”. A practical method for grading the cognitive state of patients for the clinician. J. Psychiatr. Res..

[30] Barulli M.R., Piccininni M., Brugnolo A., Musaro C., Di Dio C., Capozzo R., Tortelli R., Lucca U., Logroscino G. (2020). The Italian Version of the Test Your Memory (TYM-I): A Tool to Detect Mild Cognitive Impairment in the Clinical Setting. Front. Psychol..

[31] Miller A.S., Dykes D.D., Polesky H.F. (1988). A simple salting out procedure for extracting DNA from human nucleated cells. Nucleic Acids Res..

[32] Bai X.C., Rajendra E., Yang G., Shi Y., Scheres S.H.W. (2015). Sampling the conformational space of the catalytic subunit of human γ-secretase. eLife.

